# Knockdown of carbohydrate sulfotransferase 12 decreases the proliferation and mobility of glioblastoma cells via the WNT/β-catenin pathway

**DOI:** 10.1080/21655979.2021.1944455

**Published:** 2021-07-21

**Authors:** Juan Wang, Xiaoning Xia, Xiuqin Tao, Pingping Zhao, Chuanyu Deng

**Affiliations:** a Department of Neurology, Changle People’s Hospital, Weifang Shandong, China; b Department of Oncology, Changle People’s Hospital, Weifang Shandong, China

**Keywords:** Glioblastoma, CHST12, β-catenin pathway

## Abstract

Glioblastoma (GBM) is a common malignant tumor of the brain. Members of the carbohydrate sulfotransferase (CHST) family are deregulated in various cancer types. However, limited data are available on the role of the members of the CHST family in the development of GBM. The present study aimed to identify the role of significant members of the CHST family in GBM and explore the effects and molecular mechanisms of these significant members on GBM cell proliferation and mobility. In the current study, we demonstrated that CHST12 is the only member of CHST family that is upregulated in GBM tissues and associated with a lower survival rate according to the data obtained from The Cancer Genome Atlas. Similarly, the expression of CHST12 increased in GBM tissues than in adjacent tissues and had an important diagnostic value in distinguishing tumor tissues from adjacent tissues. The high expression of CHST12 indicated a lower overall survival rate, was negatively associated with the Karnofsky Performance Scale score, was positively associated with the KI67 expression rate, and was an independent risk factor for GBM. Knockdown of CHST12 significantly decreased GBM cell proliferation and mobility and inhibited the Wnt/β-catenin pathway. Restoration of β-catenin expression in GBM cells reversed the inhibitory effects of CHST12 knockdown on GBM cell proliferation and mobility. In conclusion, the present study demonstrated that CHST12 may be a novel biomarker for GBM; it regulates GBM cell proliferation and mobility via the WNT/β-catenin pathway.

## Graphical Abtstract

**Figure:**
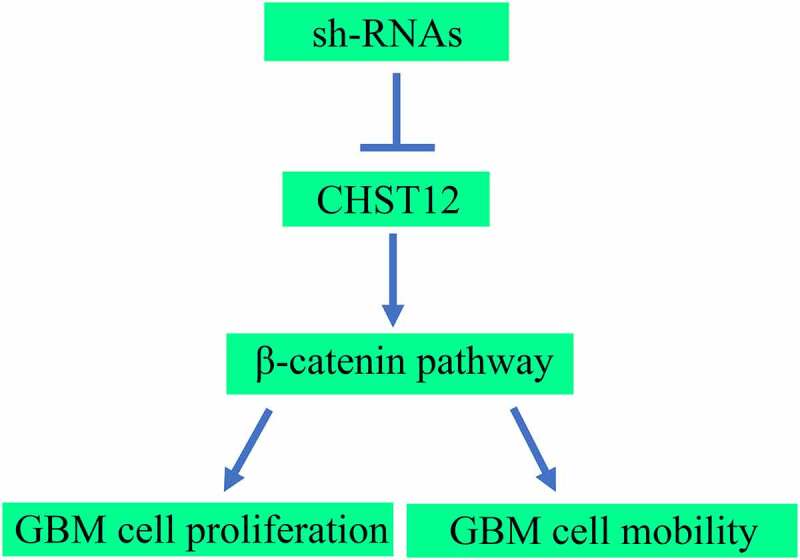

## Introduction

Glioblastoma (GBM) is a common malignant tumor in the brain tissue, with a median survival time of <24 months [1]. Resection followed by radio-chemotherapy is the main treatment method for GBM, while the median survival of GBM patients is lower than 14.6 months after undergoing the current standard treatment [2,3]. Furthermore, high levels of therapy resistance and recurrence still exist. Therefore, the exploration of novel biomarkers may help to discover drugs for GBM therapy.

Stromal proteoglycans are one of the important components of the tumor microenvironment and promote the progression of various cancer types such as lung cancer, pancreatic cancer, and GBM [4,5]. Carbohydrate sulfotransferases (CHSTs) are a class of key enzymes that contribute to tissue remodeling [6,7]. The role of the members of the CHST family in various cancers has been demonstrated in the present study. For example, the expressions of CHST11 and CHST13 increased in human hepatocellular carcinoma tissues than in adjacent tissues and promoted metastasis and drug resistance by activating the MAPK pathway [8]. The expression of CHST7 increased in the serum of lung cancer patients than that in that of healthy patients [9]. The overexpression of CHST15 in pancreatic cancer tissues is a predictor of poor outcomes [10]. CHST11 expression was increased in patients with ovarian cancer and can be used as a biomarker to predict poor prognosis [11]. CHST11 is also involved in the metastasis of breast cancer [12]. Moreover, mutations in CHST4 are correlated with a poor prognosis in patients with glioma [13]. Inhibition of CHST11 and CHST3 could potentially suppress the proliferation of glioma cell via the PI3K/AKT pathway [14]. However, the role of the members of the CHST family in GBM is largely unknown.

In the present study, we aimed to determine the significant CHST members in GBM, as well as their role and molecular mechanism in GBM cell proliferation and mobility. Bioinformatic analysis and relative biological function experiments demonstrated that CHST12 expression was increased in GBM tissues and played as an independent role in predicting poor outcomes. CHST12 knockdown decreased the GBM cell proliferation and mobility by inhibiting the Wnt/β-catenin pathway. CHST12 may be a novel and useful biomarker for the diagnosis of GBM.

## Materials and Methods

### Bioinformatics analysis

Data on the expression of CHST and the relationship between CHST family members and survival rate of GBM obtained from The Cancer Genome Atlas (TCGA) [15] were analyzed using the online tool GEPIA (http://gepia.cancer-pku.cn/) [16]. For expression analysis, adjacent normal tissues contained data from TCGA and GTEx [17], and an unpaired t-test was used to analyze the differences between adjacent normal tissues and GBM tissues. The relationship between CHST family members and survival rate was analyzed using Kaplan-Meier survival analysis. Statistical significance was set at *P* < 0.05. The expression of CHST12 in high and low EGFR amplification GBM tissues was analyzed based on the data from TCGA, while that of CHST12 in GBM tissues with wild-type and mutant IDH was analyzed based on the data from the CCGA database (http://www.cgga.org.cn/) [18]. An unpaired t-test was used to analyze the differences between these groups.

### Tissue collection

Sixty patients with GBM were enrolled in the present study. Twenty of these patients provided samples of both GBM tissues and adjacent tissues, while 40 of them only provided samples of GBM tissues. The diagnosis of GBM and adjacent tissue samples was confirmed by two independent pathologists. All patients enrolled in the present study signed a written informed consent form. The present study was approved by the Institutional Review Board of Changle People’s Hospital.

### Quantitative reverse transcription polymerase chain reaction (qRT-PCR)

Total RNA in the GBM and adjacent tissue samples was separated using the TRIzol reagent (Sigma, USA). The first cDNA was synthesized using the RevertAid RT kit (Thermo Scientific, USA). Finally, SYBR Taq (Takara, Japan) was used to determine the mRNA expression levels. The primers used in the present study were as follows: CHST12 forward primer CTTCTACTTGCACACGTCCTT and CHST12 reverse primer CTCCGTCTCCTTTCTGGGAA, CTNNB1 forward primer AAAGCGGCTGTTAGTCACTGG and CTNNB1 reverse primer CGAGTCATTGCATACTGTCCAT, and GAPDH forward primer GGAGCGAGATCCCTCCAAAAT and GAPDH reverse primer GGCTGTTGTCATACTTCTCATGG. GAPDH was set as the reference gene to determine the relative expression levels of CHST12 and CTNNB1.

### Immunohistochemistry

The samples of GBM tissues and adjacent tissues were heated at 60°C for 1 h, dewaxed using xylene, and rehydrated using descending concentrations of alcohol. Then, the tissue samples were microwaved with ethylenediaminetetraacetic acid for 30 min to retrieve the antigen. To inhibit endogenous peroxidase and decrease nonspecific binding, 0.3% H_2_O_2_ and 5% BSA were used. The samples were then incubated with anti-CHST12 antibodies (dilution 1:100; catalog number: 15,341-1-AP; Proteintech, Wuhan, China) overnight at 4°C. After washing three times with PBS, the samples were incubated with horseradish peroxidase (HRP)-conjugated secondary antibody (dilution 1:100; catalog number: BM3894; Boster, Wuhan, China) for 2 h. Finally, the samples were counterstained with hematoxylin and incubated with diaminobenzidine. The expression level of CHST12 in tissues was determined using the product of the depth of staining (0, no; 1, slight stain; 2, moderate stain; and 4, depth stain) and number of stained cells (0, <1%; 1, 1%–33%; 2, 34%–66%; and 3, >66%).

### Cell culture and transfection

GBM cell lines (U87 and LN18) were purchased from the Chinese Academy of Sciences Shanghai Branch Cell Bank (Shanghai, China). The U87 and LN18 cells were cultured in Dulbecco’s modified eagle medium (DMEM) (HyClone, USA) supplemented with 10% fetal bovine serum (FBS; Gibco, USA) in a humidified incubator at 37°C with a 5% CO_2_ atmosphere. Negative lentivirus and short hairpin RNAs (shRNAs) against CHST12 (sh-CHST12) were purchased from GeneChem (Shanghai, China). Vector and plasmid containing the CDS sequence of the CHST12 gene were purchased from GeneCopoeia (Guangzhou, China). The sequence of sh1-CHST12 was GGTTATTGATGATACTGAA, that of sh2-CHST12 was GGCAGGTGATTCTCTTCTA, and that of sh-scramble was TTCTCCGAACGTGTCACGTTT. Transfection of shRNAs and plasmids was performed using Lipofectamine 2000 (Invitrogen, USA) according to the manufacturer’s instructions. Stably transfected cells were selected using puromycin (MCE, Wuhan, China) for 14 days. The expression of CHST12 was determined by western blotting.

### Western blot

Total proteins in the cells were extracted and quantified using the bicinchoninic acid method. The samples (30 μg) were then added to each line of 10% SDS-PAGE gel (Boster, Wuhan, China) to separate and then transferred to PVDF membranes (Millipore, USA). After incubation with 5% BSA, the members were incubated with primary antibodies to CHST12 (dilution 1:1000; catalog number: 15,341-1-AP; Proteintech, Wuhan, China), β-catenin (dilution 1:1000; catalog number: 17,565-1-AP; Proteintech, Wuhan, China), E-cadherin (dilution 1:1000; catalog number: 20,874-1-AP; Proteintech, Wuhan, China), N-cadherin (dilution 1:1000; catalog number: 22,018-1-AP; Proteintech, Wuhan, China), and GAPDH (dilution 2:1000; catalog number: 60,004-1-Ig; Proteintech, Wuhan, China) overnight at 4°C. After incubation with HRP-conjugated secondary antibodies for 2 h at room temperature, the signal was developed using an enhanced chemiluminescence reagent (Boster, Wuhan, China). GAPDH was used as a loading control to determine the relative expression levels of CHST12, β-catenin, E-cadherin, and N-cadherin.

### Cell Counting Kit-8 assay

A total of 3,000 U87 and LN18 cells were injected into each well of 96-well plate. After 24 h and 48 h, a Cell Counting Kit-8 (CCK-8) reagent (YEASEN, Shanghai, China) was added to each well, and the cells were cultured at 37°C for 2 h. Finally, the absorbance (OD value) of each well was determined using a Multiskan Spectrum (Bio-Rad, USA).

### Colony formation assay

A total of 1,500 U87 and LN18 cells were injected into the cell culture dish (diameter = 6 cm) and cultured in DMEM containing 10% FBS. After culturing for 10 days, the medium was removed, and the colonies were fixed using 4% paraformaldehyde and stained with 1% crystal violet.

### Wound healing assay

The U87 and LN18 cells (1 × 10^5^) were injected into a 6-well plate and cultured until a 95% convergence was achieved. Then, a 20-μl tip was used to create a wound in the monolayer cell. After washing three times with PBS, FBS-free DMEM was added. After culturing for 0 h and 24 h, the condition of the wound was recorded. The ability of cells to migrate was assessed based on the degree of wound healing.

### Transwell assay

The 8-μm transwell chambers (Millipore, USA) with Matrigel (1:8 dilution) were used to determine the invasive ability of U87 and LN18 cells. The U87 and LN18 cells (2 × 10^4^) were resuspended in 200 μL FBS-free DMEM medium and injected into the upper chambers. Then, 700 μL of DMEM was placed in the lower chambers. After culturing at 37°C for 24 h, the medium in the upper chambers was removed. Then, the upper chambers were fixed with 4% paraformaldehyde and stained with 1% crystal violet. Finally, after removing the noninvasive cells with cotton swabs, the invasive cells in each field were examined under a light microscope (100× magnification).

### Immunofluorescence

The cells were fixed with 4% paraformaldehyde for 20 min at room temperature and then permeabilized with 0.3% Triton X-100 for 10 min. After blocking with 5% BSA, the cells were incubated with β-catenin and E-cadherin primary antibodies overnight at 4°C. Following incubation with CY3-labeled secondary antibody for 2 h, the nuclei of the cells were stained with 4′,6-diamidino-2-phenylindole for 10 min. After washing three times with PBS, the cells were examined under a fluorescence microscope (Nikon, Japan).

### Statistical analyses

All results were analyzed using SPSS (version 20.0). Quantitative variables were compared using the Student’s t-test. The relationship between CHST12 and CTNNB1 was analyzed using Pearson’s correlation analysis. The relationship between the expression of CHST12 and clinical traits was analyzed using a chi-square test. Univariate and multivariate regression analyses were used to analyze whether CHST12 was an independent prognostic factor in GBM. The diagnostic value of CHST12 was analyzed by ROC analysis. Statistical significance was set at P < 0.05.

## Results

CHST12 is the only member of the CHST family upregulated in GBM tissues and is associated with a lower survival rate according to the data obtained from TCGA. Similarly, the expression of CHST12 increased in GBM tissues than in adjacent tissues and had an important diagnostic value in distinguishing tumor tissues from adjacent tissues. The high expression of CHST12 indicated a lower overall survival rate, was negatively associated with the Karnofsky Performance Scale (KPS) score, was positively associated with the KI67 expression rate, and was an independent risk factor for GBM. Knockdown of CHST12 significantly decreased GBM cell proliferation and mobility by inhibiting the Wnt/β-catenin pathway. Restoration of β-catenin expression in GBM cells reversed the inhibitory effects of CHST12 knockdown on GBM cell proliferation and mobility.

### Role of CHST12 as a significant member of the CHST family in GBM

**Figure 1.**
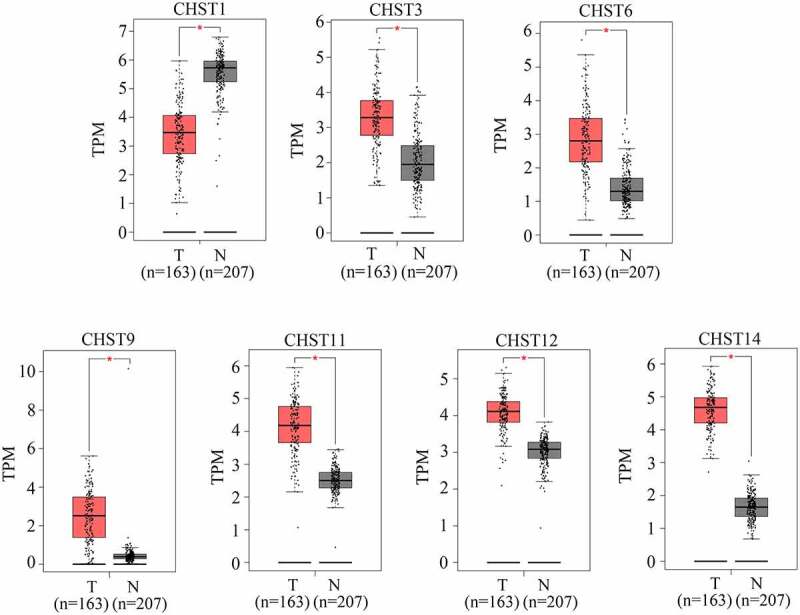
**Expression of members of the CHST family in GBM tissues and non-tumor tissues according to the data from TCGA database**. Data of 163 GBM tissues were obtained from TCGA database; the upper quartile and lower quartile of expression values in GBM tissues are shown in the red box; data of 207 non-tumor brain tissues were obtained from TCGA and GTEx. The upper quartile and lower quartile of expression values in these tissues are shown in the gray box. *P < 0.05

**Figure 2.**
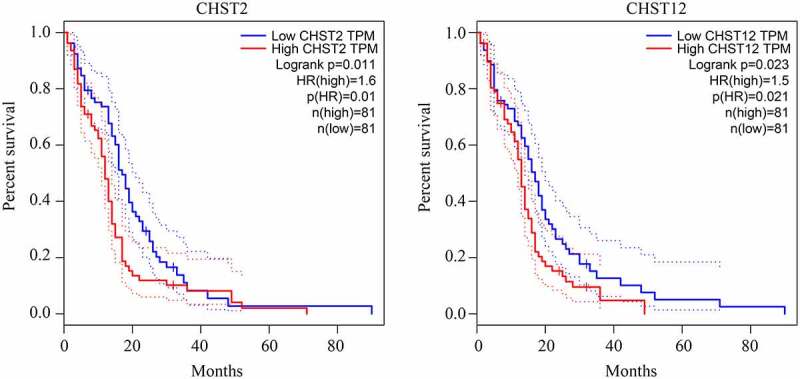
Relationship between the expression of the members of the CHST family and overall survival rate in patients with GBM

As shown in previous studies, members of the CHST family are dysregulated in various cancers. Therefore, we determined whether the members of the CHST family also played a key role in GBM. By conducting a bioinformatics analysis of the data from TCGA and GTEx, we found that CHST3, CHST6, CHST9, CHST11, CHST12, and CHST14 were highly expressed in GBM, while CHST1 expression was reduced in GBM (Figure 1). After analyzing the relationship between their expression level and survival rate in GBM, we found that the high expression of CHST2 and CHST12 was significantly associated with a lower overall survival rate (Figure 2). As CHST12 is highly expressed in GBM and associated with poor outcome, it was set as a significant member of the CHST family and enrolled in further studies.

### CHST12 highly expressed in GBM tissues and had prominent clinical value

**Figure 3.**
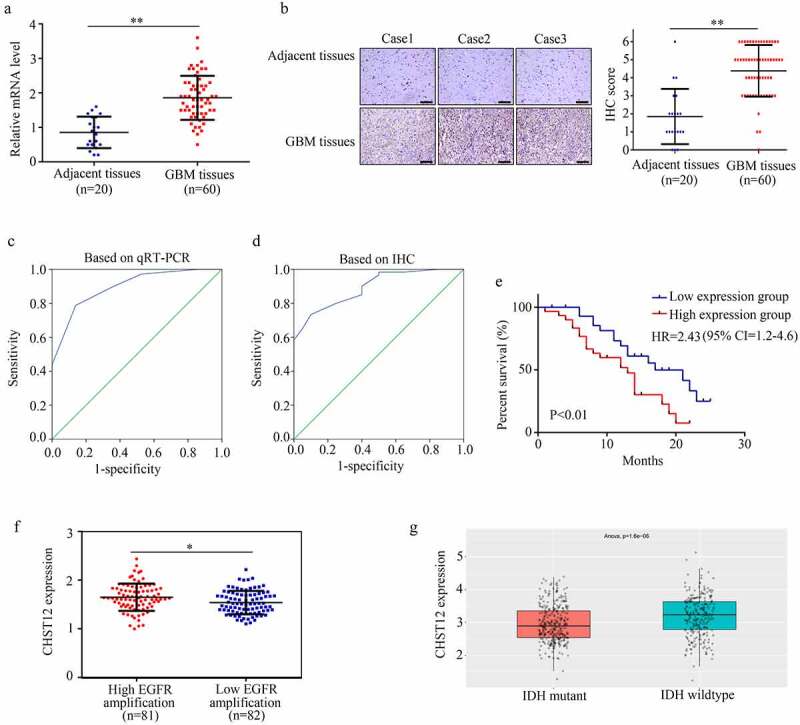
**CHST12 was upregulated in GBM tissues**. (a) qRT-PCR was used to detect the expression of CHST12 in the adjacent tissues and GBM tissues. (b) Immunohistochemistry was used to detect the expression of CHST12 in the adjacent tissues and GBM tissues. The black lines indicate a measurement of 200 μm. (c) ROC analysis of CHST12 based on qRT-PCR data. (d) ROC analysis of CHST12 based on the immunohistochemical score. (e) Kaplan survival analysis of the high expression and low expression CHST12 group. (f) The expression of CHST12 in GBM tissues with high and low EGFR amplification according to the data from TCGA database. (g) The expression of CHST12 in GBM tissues with mutant and wild-type IDH according to the data from the CGGA database. *P < 0.05; **P < 0.01

We then examined the expression of CHST12 in GBM tissues and adjacent tissues. The results showed that both mRNA (Figure 3a) and protein levels (Figure 3b) of CHST12 were highly expressed in GBM tissues than in adjacent tissues. Based on the levels of mRNA and protein detected by qRT-PCR and IHC, we performed ROC analysis. The results showed that CHST12 exhibited high diagnostic value in distinguishing GBM tissues from adjacent tissues (Figure 3c and d). According to the median expression value, GBM patients were divided into high and low expression groups; patients in the high expression group had a lower survival rate than those in the low expression group (hazard ratio = 2.43, 95% confidence interval = 1.2–4.6; Figure 3e). According to the data from TCGA and CGGA databases, CHST12 expression was also increased in GBM tissues with high EGFR amplification and wild-type IDH (figure 3f and g). Moreover, by performing the chi-square test, we found that the high expression of CHST12 was positively associated with KI67 expression in tissues and negatively associated with the patients’ KPS scores (Table 1). Furthermore, by performing univariate and multivariate regression analyses, we found that CHST12 can be an independent prognostic factor of GBM (Table 2). In conclusion, CHST12 was highly expressed in GBM tissues and could be considered to have an important clinical value.

**Table 1.** Chi-square test for analyzing the relationship between the expression of CHST12 and clinical traits of GBM patients

Parameters	CHST12			
	Low	High	X2	*P*(*)
Age			0.601	0.152
≤50	13	16		
>50	17	14		
Sex			0.271	0.181
Male	14	12		
Female	16	18		
KPS			6.696	0.008*
<80	9	19		
≥80	21	11		
Extent of resection			0.272	0.181
Subtotal resection	14	12		
Gross total resection (95%)	16	18		
Ki67			11.381	0.001*
<10%	23	10		
≥10%	7	20

**Table 2.** Univariate and multivariate regression analyses for determining the role of CHST12 in GBM

	Univariate analysis			Multivariate analysis		
Parameters	HR	95% CI	*P*(*)	HR	95% CI	P(*)
Age	1.031	0.972–1.098	0.563	– –	– –	– –
Gender (female vs. male)	0.934	0.737–1.23	0.628	– –	– –	– –
KPS score	0.773	0.628–0.873	0.034	0.944	0.878–1.043	0.187
Radiation (true vs. false)	0.335	0.213–0.534	0.001*	0.428	0.313–0.872	0.043*
Temozolomide chemotherapy (true vs. false)	0.393	0.229–0.477	0.001*	0.567	0.442–0.686	0.008*
CHST12 expression	1.335	1.119–1.521	0.003*	1.298	1.089–1.373	0.021*

### Knockdown of CHST12 significantly decreased the GBM cell proliferation

**Figure 4.**
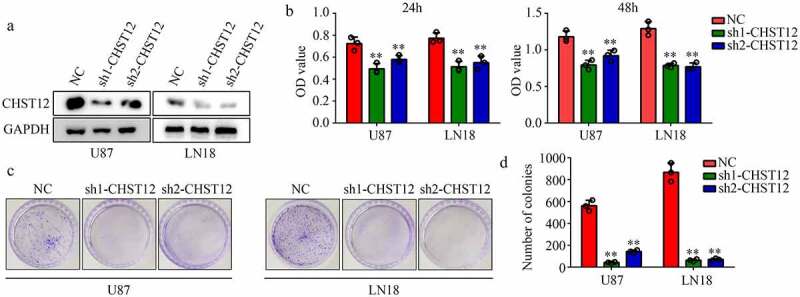
**Knockdown of CHST12 decreased the proliferation of GBM cells**. (a) Western blot was used to detect the expression of CHST12 in the CHST12 knockdown group and normal control (NC) group. (b) CCK-8 was used to detect the proliferation of the CHST12 knockdown group and NC group. (c) Colony formation assay was used to detect the colony formation ability of the CHST12 knockdown group and NC group. **P < 0.01

Furthermore, we used targeting CHST12 shRNAs to construct CHST12 with low expression of U87 and LN18 GBM cell lines (Figure 4a). The CCK-8 results showed that the inhibition of CHST12 decreased the proliferation of both U87 and LN18 cells at 24 h and 48 h (Figure 4b). Similarly, the suppression of CHST12 expression inhibited the colony formation ability of both U87 and LN18 cells (Figure 4c–D).

### Suppression of CHST12 expression inhibited GBM cell mobility

**Figure 5.**
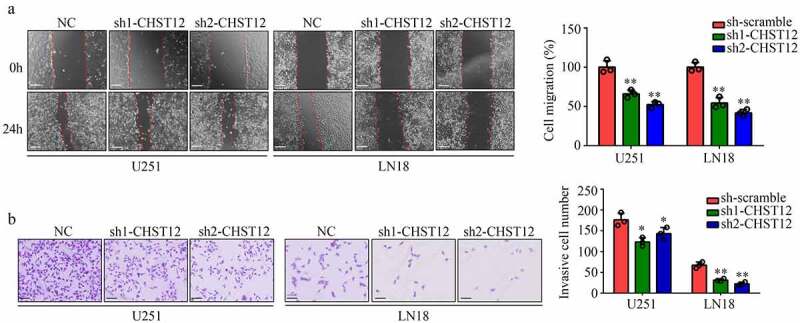
**Knockdown of CHST12 inhibited the mobility of GBM cells**. (a) Wound healing assay was used to detect the migration of CHST12 knockdown group and negative control (NC) group. The white line indicates a measurement of 250 μm. (b) Transwell assay was used to detect the invasion of CHST12 knockdown group and NC group. The black lines indicate a measurement of 100 μm.*P < 0.05; **P < 0.01

A wound healing assay was performed to determine the migration of cells transfected with shRNAs targeting CHST12. The results showed that U87 and LN18 cells with low CHST12 expression exhibited lower migration rates (Figure 5a). Moreover, transwell assays indicated that the suppression of CHST12 expression obviously inhibited the invasion of U87 and LN18 cells (Figure 5b). Taken together, the suppression of CHST12 expression obviously inhibited the GBM cell mobility.

### Inhibition of CHST12 significantly decreased the WNT/β-catenin pathway in GBM cells

**Figure 6.**
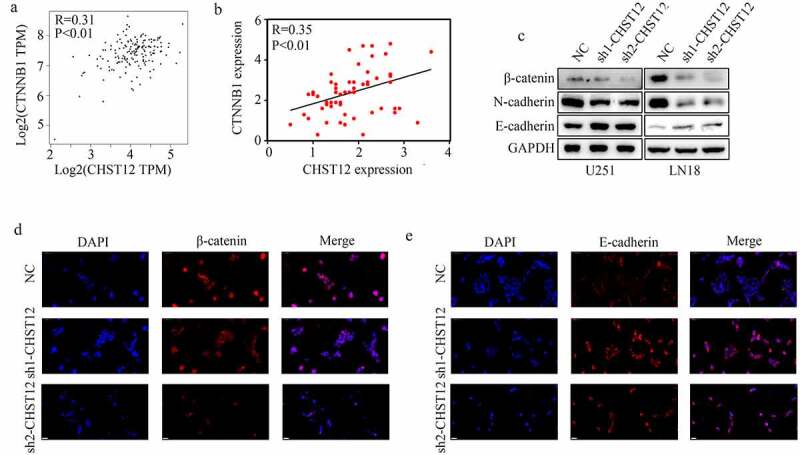
**Knockdown of CHST12 inhibited the activation of WNT/β-catenin pathway**. (a-b) The co-expression relationship between CHST12 and CTNNB1 in our GBM tissues and TCGA GBM tissues. (c) The expression of β-catenin, E-cadherin, and N-cadherin in the negative control group and CHST12 knockdown groups. (d–e) Immunofluorescent staining detected the expression of β-catenin and E-cadherin in the negative control group and CHST12 knockdown groups. The white lines indicate a measurement of 50 μm

CHST12 was co-expressed with β-catenin (also named CTNNB1) in TCGA GBM tissues (Figure 6a) and GBM tissues from our research group (Figure 6b). To further verify the relationship between CHST12 and the WNT/β-catenin pathway, western blotting was performed. The results indicated that the inhibition of CHST12 significantly decreased the expression of β-catenin, decreased the expression of N-cadherin, and increased the expression of E-cadherin (Figure 6c). Moreover, immunofluorescence showed that inhibition of CHST12 decreased the expressions of nuclear and cytoplasmic β-catenin (Figure 6d) and increased the expression of E-cadherin (Figure 6e). Taken together, these results indicated that the inhibition of CHST12 expression significantly decreased the WNT/β-catenin pathway in GBM cells.

### Restoration of the β-catenin expression reversed the effects of CHST12 knockdown

**Figure 7.**
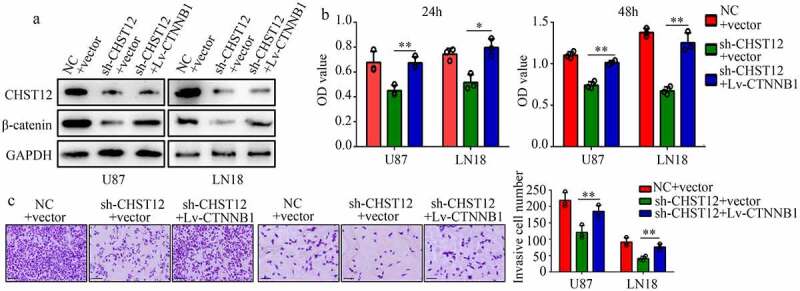
**Restoration of the β-catenin expression reversed the effects of CHST12 knockdown**. The GBM cells were divided into three groups: negative control (NC), CHST12 knockdown (sh-CHST12), and sh-CHST12 + β-catenin overexpression (Lv-β-catenin). (a) Western blot was used to detect the expression of β-catenin and CHST12 in each group. (b) CCK-8 was used to detect the proliferation in each group. (c) Transwell assay was used to detect invasion in each group. The black lines indicate a measurement of 100 μm. *P < 0.05; **P < 0.01

To determine whether the WNT/β-catenin pathway is involved in the biological function induced by CHST12, we restored the expression of β-catenin in CHST12 knockdown cells using sh1-CHST12 and β-catenin plasmid (Figure 7a). The results of CCK-8 assays showed that restoration of β-catenin expression significantly relieved the inhibitory effects of CHST12 knockdown on U87 and LN18 cell proliferation (Figure 7b). Moreover, the results of transwell assays showed that increased β-catenin expression reversed the inhibitory effects of CHST12 knockdown on U87 and LN18 cell invasion (Figure 7c).

## Discussion

Various studies have indicated that GBM is challenging for clinicians because of its rapid and infiltrative growth, leading to invasion in the normal brain tissues and resulting in incomplete resection and recurrence [19]. Therefore, identification of the specific oncogenes that regulate the proliferation and invasion of GBM has contributed to development of effective GBM therapy.

CHSTs are a class of key enzymes that can catalyze the transfer of sulfate to position 4 of the N-acetylgalactosamine (GalNAc) residue of chondroitin and desulfated dermatan sulfate and play a key role in carbohydrate metabolism [7]. Several members of the CHST family have been reported to be oncogenes in various types of cancers. CHST2 and CHST7 were used as biomarkers for pediatric high-risk B-precursor acute lymphoblastic leukemia, and patients with high CHST2 and CHST7 expression had poor 4-year relapse-free survival rates [20]. CHST4 was identified as an independent prognostic factor in patients with HBV-HCC [21]. CHST11 and CHST13 were highly expressed in HCC tissues, and knockdown of CHST11 and CHST13 can decrease HCC cell metastasis and drug sensitivity *in vivo* [8]. Furthermore, CHST11 and CHST13 have been reported as oncogenes in gliomas [14].

Based on the fact that the members of the CHST family plays a key role in various types of cancers, we identified the significant members of CHST family causing GBM. Through a bioinformatics analysis, we found that among the members of the CHST family, CHST12 was upregulated in GBM and high expression of CHST12 was a predictor of poor outcomes in patients with GBM. Therefore, we focused on investigating the role of CHST12 expression in GBM. CHST12, a member of the CHST family, is mainly localized in the Golgi membrane in normal cells and catalyzes the transfer of sulfate to position 4 of the GalNAc residue of chondroitin and desulfated dermatan sulfate [22]. Reduction in the CHST12 expression was reported in adults with osteoarthritis, while inhibition of CHST12 promoted inflammation [23,24]. Dysregulation of CHST12 is involved in the occurrence of renal damage in patients with lupus [25]. Furthermore, the high expression of CHST12 was positively associated with the progression of brain diseases, such as multiple sclerosis [26]. Based on this finding, we further explored the role of CHST12 in GBM. We then analyzed the expression of CHST12 in GBM tissues from our research group. We found that both mRNA and protein levels of CHST12 were upregulated in GBM tissues than in adjacent tissues, and CHST12 has remarkable value in distinguishing GBM tissues from adjacent tissues. High CHST12 expression was considered a predictor of poor outcomes. Because CHST12 and EGFR (a major oncogene in GBM) were both localized in chromosome 7 [27], we analyzed the relationship between CHST12 and EGFR and found that CHST12 expression was increased in GBM tissues with high EGFR amplification. Based on this evidence, we speculated that CHST12 may be involved in the rapid proliferation of GBM cells. Consistent with this speculation, we analyzed the relationship between CHST12 expression and clinical traits and found that high expression of CHST12 was positively associated with KI67 expression (a biomarker for quick proliferation) in tissues and negatively associated with the KPS score. Moreover, CHST12 could be an independent prognostic factor for GBM. GBM patients with IDH mutations are more likely to have a low Ki-67 expression level [28]. We found also that CHST12 expression was higher in patients with wild-type IDH. This may be another reason for the association between CHST12 and higher Ki-67 expression in GBM tissues. Moreover, as this evidence supports that EGFR amplification, KI67 expression, and IDH status are widely used biomarkers for predicting the outcome of GBM patients [29] and CHST12 has the potential to link them, we considered that CHST12 may have significant clinical value in the development of GBM. Furthermore, we found that inhibition of CHST12 significantly decreased GBM cell proliferation and mobility. All these evidences indicated that CHST12 may be a novel biomarker and targets for the GBM diagnosis and therapy.

To explore the molecular mechanism of CHST12, we analyzed the GBM data from TCGA and found that CHST12 expression was associated with β-catenin expression. Moreover, CHST12 co-expressed β-catenin in GBM tissues from our research group. The Wnt/β-catenin pathway is abnormally activated in GBM tissues [30]. After receiving the upstream activation signal, β-catenin, the core protein of the WNT/β-catenin pathway, is translocated into the nucleus, thus affecting the expression of downstream genes, such as E-cadherin and N-cadherin [31,32]. To verify whether CHST12 affects the WNT/β-catenin pathway in GBM, relevant molecular biology experiments were performed. We showed that knockdown of CHST12 significantly inhibited the WNT/β-catenin pathway. Restoration of β-catenin expression in GBM can reverse the inhibitory effects of CHST12 knockdown on cell proliferation and mobility. These data provide the first evidence to support that knockdown of CHST12 may be a therapeutic strategy to inhibit the activation of the WNT/β-catenin pathway in GBM.

Actually, there are some limitations in the present study. First, adherent GBM cell lines are poor cellular models for invasion as they have limited invasive potential *in vivo*. Therefore, we did not perform *in vivo* invasion model to detect the effects of CHST12 on GBM cell invasion in vivo. Moreover, the pro-proliferation effects of CHST12 *in vivo* was also not detected. Therefore, more experiments should be further performed to determine the role and molecular mechanism of CHST12 in GBM.

## Conclusion

Taken together, we found that CHST12 is a significant member of the CHST family in GBM, which is highly expressed and has remarkable diagnostic and clinical value. Knockdown of CHST12 decreases the proliferation and mobility of GBM cells via the Wnt/β-catenin pathway. CHST12 may be a novel biomarker and a potential target for GBM treatment.

## Data Availability

The data used for analyzing the expression of members of the CHST family and their relationship with survival rate can be obtained from the GEPIA database (http://gepia.cancer-pku.cn/). Relative experimental data can be obtained from the corresponding author on reasonable request.

## Highlights

(1) CHST12 was up-regulated in GBM tissues, and predicted poor outcome.

(2) CHST12 was associated with high KI67 expression and lower KPS score in GBM.

(3) Inhibition of CHST12 decreased the proliferation and mobility of GBM cells *in vitro*.

(4) Knockdown of CHST12 inhibited the activation of WNT/β-catenin pathway.

(5) Restored β-catenin expression reversed the effects of CHST12 knockdown.
